# Universal Full-Day Kindergarten and Maternal Labor Supply: A Life-Cycle Analysis

**DOI:** 10.21203/rs.3.rs-4550789/v1

**Published:** 2024-06-26

**Authors:** Ashley Erceg, Katie Genadek, Kristine West

**Affiliations:** a Center for Economic Studies, United States Census Bureau;; b Contact Author: University of Colorado - Boulder, IZA; Institute of Behavioral Science, UCB 483, University of Colorado, Boulder, CO, USA 80309-0401;; c St. Catherine University; 2004 Randolph Ave, St Paul, MN, USA 55105;

**Keywords:** labor supply, mothers, public schooling, childcare subsidies, J13, J18, J20, J30, I2

## Abstract

We estimate the effect of state-level policies enacting universal free full-day kindergarten on mothers’ labor supply using a life-cycle analysis. Similar to previous research on childcare and labor supply, we find that free full-day kindergarten increases labor force participation rates for mothers whose youngest child is kindergarten-aged by 4.3 to 7.1 percentage points. We find that for mothers whose youngest child is an infant, labor force participation increases by 7.2 to 9.8 percentage points, and for women whose youngest child is 3 to 4 years old labor force participation increases by 5.9 to 7.9 percentage points. The fact that the policies impact the labor supply for mothers of younger-than-kindergarten-age children by even more than for mothers of kindergarten-aged children is important for understanding the full effect of subsidized childcare. This is consistent with a life-cycle model of labor supply where wages and prices in future periods impact mothers’ labor force attachment.

The main policy goal of kindergarten expansion is to invest in children’s human capital, however, it also acts as a large childcare subsidy for parents of young children. Access to affordable care for children is an important determinant in a woman’s labor supply (Anderson & Levine, 1999; Baum, 2003; Blau & Tekin, 2003). The COVID-19 pandemic has only highlighted the importance of access to care for women with young children. Over one quarter of all employment loss due to COVID-19 was for women with children under age six, likely due to care constraints (Pitts, 2021). Expansions to public schooling, including additional years of schooling (kindergarten/pre-kindergarten) or more hours of schooling (extended school days), should yield both learning gains for children and additional labor market participation for parents, especially mothers. Research on the effect of schooling on women’s labor supply in the United States has been mixed (Halim, Perova, and Reynolds, 2023). Some evidence suggests a negligible effect on mothers’ employment (Fitzpatrick, 2012), while other research finds gains for single and lower-income mothers (Casico, 2009). We build on this previous literature by investigating changes in maternal labor supply, on both the extensive and intensive margins, caused by the expansion of full-day kindergarten in the United States.

We look at state-wide adoption of universal free full-day kindergarten because full-day kindergarten aligns with workdays better than part-day kindergarten. We employ a triple difference-in-differences design to compare employment outcomes of mothers with young children to non-mothers and mothers whose youngest child is too old for kindergarten, before and after the introduction of universal free full-day kindergarten, in states that do and do not adopt universal full-day kindergarten.

We use data from the Current Population Surveys (CPS) and the US Decennial Censuses combined with the American Community Survey (ACS), covering 1970 to 2015. We find that state-wide free full-day kindergarten increases maternal labor force participation, and, interestingly, the impact is largest for mothers with whose youngest child is still too young to be eligible for kindergarten. The results are consistent with a life-cycle model of labor supply (Smith 1977, Heckman and Macurdy 1980, Kimmel and Kniesner 1998, Bailey 2006) where the decision to return to work is made when a child is an infant or toddler rather than when a child is school-aged. A life-cycle or multiperiod maximization of lifetime earnings accounts for expected future childcare costs and expected future income. Within the multi-period framework, the adoption of free full-day kindergarten will have the largest impact on women who are deciding whether to return to the labor market shortly after the birth of a child. This aspect of early education policy has been overlooked by previous research and is policy relevant.

Previous literature has found mixed results when analyzing the effect of public kindergarten and pre-kindergarten on maternal labor force participation in the United States (Gelbach 2002; Baker et al. 2008; Casico 2009; Fitzpatrick 2010; Havnes & Mogstad 2011; Cascio & Schanzenbach 2013; Sall 2013; Gibbs 2014; Bauernschuester & Schlotter 2015; Hardoy and Schone 2015, Li 2020). The mixed conclusions of previous research are likely the result of focusing on only mothers with eligible children, rather than investigating the impacts of expanded schooling on mothers with children of all ages. While mothers of eligible children are the most obvious beneficiaries of the childcare subsidy implicit in public schooling, mothers of kindergarten-aged children have, in large part, already made their decisions about work. Mothers of infants, on the other hand, while only future beneficiaries of publicly provided kindergarten, are making key decisions about labor force attachment, and thus policy changes are more likely to have an impact on them. A life-cycle model of labor supply, rather than a single-period labor-leisure model, better describes this reality.

## I. Kindergarten and the life-cycle model of labor supply

The cost of caring for children, especially young children is an important factor in labor force participation decisions (Blau & Robins 1988; Connelly 1992; Kimmel 1998; Powell 2002). Public schooling dramatically decreases the cost of childcare; thus, we expect expansions in public school availability to impact mothers’ labor supply. Previous work has focused on the impact on mothers who are immediate beneficiaries of kindergarten’s implicit childcare subsidy. We look at mothers who are direct beneficiaries as well as those who are future beneficiaries and thus may make different labor force participation decisions in anticipation of decreased childcare costs when their child reaches kindergarten age.

Full-day kindergarten more closely aligns with the workday, reducing the cost of childcare a mother would pay, and thus increasing her take-home wage.^1^ A one-period labor-leisure model suggests that the impact of the adoption of free full-day kindergarten will be largest for mothers whose youngest child is kindergarten aged because only mothers whose youngest child is in kindergarten see their take-home wage directly impacted by the introduction of full-day kindergarten. Mothers whose youngest child is not yet kindergarten-aged see no change in their wages since they will still have to pay for childcare if they return to work. Likewise, the wage for mothers whose youngest child is past kindergarten is unchanged since they already have the option of free full-day school-based childcare.

This one-period labor-leisure model, however, misses mothers’ key labor force participation decisions, which are not made when their youngest child enters school, but rather when their children are infants. Labor force decisions are most germane, and thus policy impacts will be most keenly felt for mothers of infants for at least two reasons: (1) Women who take substantial time away from the labor market have trouble reentering the labor markets and experience lower wages throughout their careers (Ruhm 1998; Waldfogel 1997; Lundberg and Rose 2000; Anderson et al. 2003). Time away from the labor force comes with a substantial wage penalty and mothers of infants have a longer time horizon for which to bear or avoid this reality; (2) The relationship between time away and the resulting wage penalty is likely non-linear. More research is needed on the exact functional form, but it likely has a stepwise nature (i.e. 0.5 years out of the labor force may not be all that different from 1 year but quite different from 1.5 years).^2^ Once a mother has left the workforce for a substantial period of time, she has sent a strong signal to potential future employers that she is the lead caregiver rather than the lead earner in her household. The employer is thus more likely to put her on “the mommy track” and she will incur a larger motherhood wage penalty than a mother who returned more quickly after the birth of a child (Schwartz 1989).

Taken together these facts suggest that mothers of younger children are more likely to be impacted by the introduction of free full-day kindergarten. Mothers of infants have to decide if they are going to take time away from the labor force while mothers of kindergarten-aged children have already made this decision.^3^ Kindergarten reduces or eliminates the cost of childcare when a child is five or six years old. By this time, a mother who has chosen to “stay at home” and engage in full-time home production and childcare has been out of the workforce for at least five years. Indeed, it is likely longer given that a kindergarten-aged child may have older siblings and the mother may have left the labor force at the birth of her first child.

To accurately model mothers’ work decisions we need to use a life-cycle approach that allows for intertemporal substitution of labor and future wages due to a decrease in the costs of childcare (Smith 1977, Heckman and Macurdy 1980, Kimmel and Kniesner 1998, Bailey 2006). The life-cycle model asserts that current labor supply depends on wages and childcare prices in future periods.

## II. Previous Literature

The existing literature on the impact of kindergarten on maternal labor supply yields mixed results with the primary impact on only single mothers and women with less than a baccalaureate education. Most notably, Gelbach (2002) investigated the impact of half-day kindergarten on maternal labor force participation using quarter of birth to instrument for age of youngest child and 1980 Decennial Census data. He finds that, after the introduction of half-day kindergarten, single women whose youngest child is age five increased labor force participation by 6 percent. Casico (2009) used a difference-in-difference design and U.S. decennial census data spanning 1950–1990. She finds that single women whose youngest child is five work 0.05 weeks and 2.6 hours more after the introduction of half-day kindergarten.

In a related literature, investigations into the relationship between universal childcare or pre-kindergarten and female labor supply have also produced mixed results. While some research has shown universal or subsidized childcare increased maternal labor supply (Baker et al. 2008; Sall 2013; Bauernschuester & Schlotter 2015; Hardoy and Schone 2015, Boll & Lagemann 2019, Eckhoff Andresen & Havnes 2019, Davis, Carlin, Krafft & Forry 2019, Muller & Wroholich 2020), other work has shown no or limited impact on maternal employment (Fitzpatrick 2010; Havnes & Mogstad 2011; Cascio & Schanzenbach 2013, Li 2020). Similar to the research on kindergarten, some of the studies on subsidized childcare suggest that there is only an impact on labor supply for low-income or single mothers (Goux & Marin 2010; Fitzpatrick 2012). Like the previous research on kindergarten, most of this literature has focused on mothers who are the immediate and direct beneficiaries of the policy rather than mothers with younger children who are future beneficiaries and often does not consider alignment of childcare with the workday.

In addition to focusing on mothers with kindergarten-aged children (and ignoring mothers of younger children), previous research on kindergarten adoption in the United States has primarily focused on the impact of half-day kindergarten (e.g. Gelbach 2002, Casico 2009). The timing of full-day kindergarten should have a larger impact on maternal employment than half-day kindergarten because full-day kindergarten aligns more closely with working hours (Ruppanner, Moller & Sayer 2019, Gambaro, Marcus & Peter, 2019). Moreover, half-day kindergarten leaves a large need for childcare as families must coordinate either parental or paid childcare for a larger portion of the day. Some school districts offer the possibility to enroll in full-day programming, but parents must pay for the full-day option so, in all cases, half-day kindergarten will reduce, but not eliminate, the cost of childcare. We are aware of only two other papers that investigate the transition from half-day to full-day kindergarten. Gibbs (2014b) uses data from Indiana and Dhuey, Eid & Neill (2020) use data from Ontario, Canada to analyze local adoption of full-day kindergarten and both find some evidence that women’s employment increased and estimates were strongest for single mothers. We add to this literature by investigating state-level universal free full-day kindergarten adoption using nationally representative data from the CPS and the decennial censuses.

Research from other country contexts has found that increasing school day length increases women’s labor supply. Padillia-Romo & Cabrera- Hernández (2019) use panel data from Mexico to examine full-time school program that extended the school day from 4.5 to 8 hours in elementary schools. They find that the extension increased labor force participation, hours worked, and earnings among mothers. Berthelon, Kruger, & Oyarzún (2022) uses panel data from Chile to examine the effect of a reform which increased schooling from half-day to full day (an average increase between 1.5 and 2 hours per day). Lengthening the school day increase women’s labor force participation, employment, and hours worked. We contribute to this literature by examining the effect of school day extensions in the United States.

## III. Full-day Kindergarten in the United States

Table 1 shows the years in which states enacted legislation for universal free full-day kindergarten. For the purpose of this study we define full-day kindergarten as being five hours or more per day. This is nearly double the typical time of half-day kindergarten.^4^ The first state to adopt full-day kindergarten was Alabama in 1973. The District of Columbia and North Carolina both adopted full-day kindergarten in 1978 and other states followed slowly in the 1980s and 1990s. More recently, Oklahoma adopted full-day kindergarten in 2013.^5^ Notably all the states that adopt full-day kindergarten during the period studied are southern or mid-Atlantic states.

It is important to note that universal free full-day kindergarten is a subset of all full-day kindergarten in the United States. In many states, some districts offer free full-day kindergarten, in other districts, full-day kindergarten is available for a fee but that fee is waived for low-income families. Full-day kindergarten can be quite widespread in states without a state mandate. For instance, Gibbs (2014a, 2014b) studies the roll-out of full-day kindergarten in Indiana. Indiana is not included in Table 1 because full-day kindergarten is not a statewide policy.^6^ Given this, our estimates should be interpreted as the effect of a *state mandate* for funded universal full-day kindergarten on maternal employment.^7^

For two reasons we expect that our estimates are a lower bound. First, states that do not have statewide full-day kindergarten have at least some districts that offer free full-day kindergarten to all or some of their families, and thus, the control group experiences a non-zero level of the treatment. The comparison is not between half-day kindergarten and full-day kindergarten but rather between the average mix of half/full-day kindergarten and universal free full-day kindergarten. Second, statewide full-day kindergarten could be endogenous to the spread of full-day kindergarten at the district level within a state.

## IV. Data

We use pooled cross-sectional data spanning 1970–2015 from two nationally representative surveys for our analysis. For our main analysis we use microdata from the Current Population Survey (CPS) Annual Social and Economic (ASEC) Supplement obtained from IPUMS-CPS, from 1978–2015 (Flood et al. 2015). The ASEC is the largest supplement to the CPS, which is a monthly survey and primary source of labor force information for the Bureau of Labor Statistics (BLS). The ASEC questionnaire, given to all respondents in March as well as some respondents in February, April, and November, includes more information on employment and education than the basic monthly questionnaire. The CPS data are useful for the analysis because they have yearly data and they have consistent measures of weekly hours worked and hours worked per year making it preferable for the intensive margin analysis. We examine the robustness of our results in various ways, including using the public use microdata samples (PUMS) from the U.S. decennial censuses and the American Community Survey (ACS) obtained from IPUMS-USA, 1970–2015 (Ruggles et al. 2015).^8^ For 1970–2000 we use the PUMS samples from the decennial census, and we use ACS data from 2005, 2010, and 2015.^9^

Table 2 provides summary statistics for our analytic samples. We limit our analysis to women between the ages of 18 and 55. This age range includes prime-age workers who are most likely to have a young child at home.^10^ The average age in both data sets and for women in state-years with and without universal full-day kindergarten is just under 36. We show averages for both women in state-years with universal full-day kindergarten and women in state-years without universal full-day kindergarten. Other demographic characteristics vary little between women in state-years with and without universal full-day kindergarten. The differences that are statistically significant are all consistent with the adoption of free full-day kindergarten primarily occurring in southern states. For instance, women in full-day kindergarten state-years are more likely to be non-white. Women in full-day kindergarten state-years have slightly lower average incomes and are less likely to have college degrees. All of these variables are used as controls in the analysis.

## V. Method

Full-day kindergarten laws were introduced at different times across states; thus they generate a useful quasi-experiment for examining the effects on maternal labor force participation. In our analysis, we compare mothers in states with and without universal full-day kindergarten to women without school-aged children in states with and without universal full-day kindergarten from 1970–2015.^11^ Before turning to this triple-difference model, we start by examining the change in labor force participation using a simple difference-in-differences model, which compares the average labor supply of women, in states-year with universal full-day kindergarten to women in states without universal full-day kindergarten. We estimate the following model given by equation 1:

$$Y_{ist}=\alpha +\theta C_{ist}+\delta FDK_{st}++\beta_{s}I_{s}+\gamma_{t}I_{t}+\tau X_{ist}+\varepsilon_{ist}$$

The outcome, *Y*, is a measure of labor force participation for respondent *i* in state *s* and year *t*. Our primary results focus on a simple binary indicator that takes the value 1 if the woman is in the labor force (employed or unemployed) and 0 if the woman is not in the labor force (engaged in full time home production). We estimate linear probability models (LPM) using Ordinary Least Squares (OLS) regression to estimate the probability of a woman choosing to be in the labor force. Our coefficient of interest, *FDK*_*st*_, reports the average percentage point change in labor force participation for women living in states with universal full-day kindergarten.

Next, we implement a triple difference analysis. The treatment group is comprised of mothers with young children in state-years with universal full-day kindergarten and the comparison groups are non-mothers as well as mothers with older children in those state-years as well as all women in state-years without universal full-day kindergarten.

The triple difference (DDD) method is described by Equation 2:

$$Y_{ist}=\alpha +\theta C_{ist}+\delta FDK_{st}+\varphi \left(FDK_{st}*C_{ist}\right)+\beta_{s}I_{s}+\gamma_{t}I_{t}+\tau X_{ist}+\varepsilon_{ist}$$

The outcome, *Y*, is a measure of labor force participation for respondent *i* in state *s* and year *t*. Our primary results focus on a simple binary indicator that takes the value 1 if the woman is in the labor force (employed or unemployed) and 0 if the woman is not in the labor force (engaged in full time home production). We estimate linear probability models (LPM) using Ordinary Least Squares (OLS) regression to estimate the probability of a woman choosing to be in the labor force.

We also estimate the number of hours of work last year, a measure of the intensive margin of labor supply, for those working. We construct hours worked last year by multiplying reported weeks worked last year and usual hours worked per week last year. These two measures are consistent and exact in the CPS data, but in the decennial census and ACS data, the measures vary by sample. For all years except for 1970, we use usual hours worked per week, but for 1970 respondents were only asked hours worked last week. We use the midpoints of intervals reported for weeks worked last year for all years, because the majority of years in this data only have intervals reported.

Equation 1 includes an indicator for living in a state with universal full-day kindergarten (FDK_st_) and having a youngest child that is kindergarten aged (C_ist_). With the inclusion of state and year fixed effects (I_s_, I_t_,), the interaction between FDK_st_ and C_ist_ is essentially a triple interaction and the intent-to-treat DDD estimate. The DDD strategy addresses a number of potentially important endogeneity issues. Women’s labor supply decisions are strongly influenced by the presence of children and fertility decisions are likewise influenced by labor force decisions. Similarly, fertility and childcare are also simultaneously determined. The anticipation of childcare costs is likely a factor in the choice to have children and how many children to have. The DDD estimator helps to mitigate this endogeneity because it allows for comparisons of labor force participation of mothers with young children and women without young children in states with and without exogenous changes in childcare costs due to the introduction of universal free full-day kindergarten.

By using multiple years of data, the DDD estimator also controls for the potential endogeneity of the laws themselves. If there are permanent differences in the characteristics of states, the differencing will account for these by only looking at *changes* in labor force participation rather than *levels*. If mothers are more likely to work in full-day kindergarten states regardless of full-day kindergarten laws, the DDD estimator addresses this problem. The remaining problem would only be if full-day kindergarten states were experiencing differential *trends* in labor force participation, i.e. maternal labor force participation was increasing at a faster rate in full-day kindergarten states prior to the adoption of full-day kindergarten laws and may have continued to increase at a faster rate regardless of the law change.

**Figure 1.**
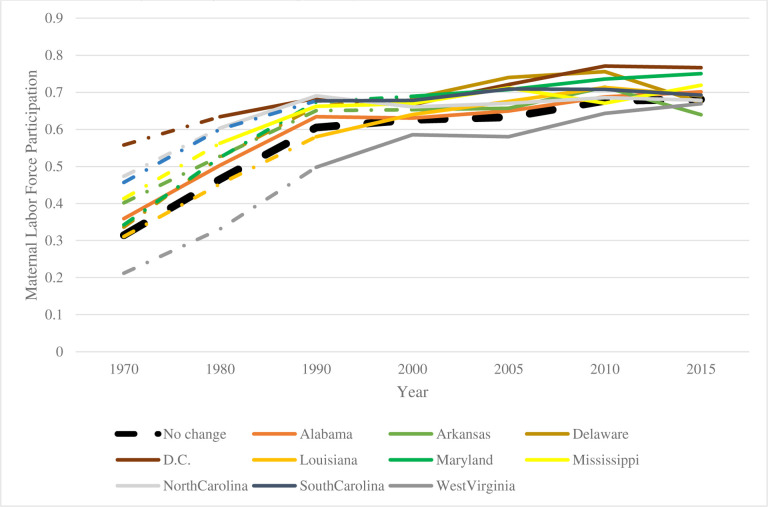
Maternal Labor Force Participation for Women with Children under Age 6, Pre and Post Free Full-Day Kindergarten Adoption by State, U.S. Decennial Census Data

Figure 1 below provides a visual representation of maternal labor force participation trends in states that do and do not adopt full-day kindergarten. The dashed portion of the lines are in the period prior to adoption and the solid portion of the lines is post-adoption. The black dashed line is the control states that never adopt. We see no evidence that the states that adopt full-day kindergarten were on differential labor force participation trends prior to adoption, however, equation 1 allows for a more formal test and control for this possibility.

The X in Equation 1 represents a vector of individual and household characteristics. In this vector, we include the woman’s age (a linear and a quadratic term as well as an indicator for women who are traditional college age, 18–22), non-labor income (a linear and a quadratic term), indicators for marital status, race and ethnicity, education, urban residence, as well as the number of household children and the number of household adults. Controlling for time variant changes in these observable characteristics strengthens the DDD analysis.

The main coefficient on full-day kindergarten (δ) estimates the difference in labor force participation between women *without* kindergarten-aged children after the adoption of full-day kindergarten in states that adopt relative to states that do not adopt. These may be women with children younger than five, mothers of children older than six, or women without children. As described above, this follows previous literature in this area by focusing on mothers with kindergarten-aged children and thus can immediately take advantage of the change in policy.

The advantage of focusing on mothers who are direct beneficiaries is that it narrows and clarifies the treatment group. This alleviates concerns about contemporaneous shocks that may confound results. For instance, if states are likely to introduce full-day kindergarten at the same time as they increase funding for Head Start or other pre-K programming, it would be impossible to say if changes in labor force participation for mothers with pre-K aged children is a result of the pre-K programming or the anticipation of full-day kindergarten. This is a particular concern in single-state studies. For national data, such as the CPS or the decennial census used in this study, the problem would have to be that all states enact full-day kindergarten at the same time as they implement other policies that also influence mothers’ labor force participation decisions. This is certainly possible, however, that narrowing the definition of the treatment group to only include current beneficiaries also has disadvantages. Most importantly, it may miss life-cycle impacts, and ignoring these impacts could lead policymakers to underestimate the impact of full-day kindergarten on maternal labor supply.

In order to capture the life-cycle impacts of full-day kindergarten adoption, we expand the model to include indicators for the age of the youngest child as a categorical variable. This also allows us to treat the age of youngest child non-parametrically. If we had a single, continuous variable, we would force a linear relationship that may not be appropriate. We categorize youngest child as infant (0–2), preschool age (3–4), and kindergarten age (5–6). Thus, our preferred econometric model is given by Equation 3:

$$Y_{ist}=\alpha +\delta FDK_{st}+\varphi_{02}\left(FDK_{st}*C_{ist}\left(0\_2\right)\right)+\varphi_{34}\left(FDK_{st}*C_{ist}\left(3\_4\right)\right)+\varphi_{56}\left(FDK_{st}*C_{ist}\left(5\_6\right)\right)+\theta_{02}C_{ist}\left(0\_2\right)+\theta_{34}C_{ist}\left(3\_4\right)+\theta_{56}C_{ist}\left(5\_6\right)+\beta_{s}I_{s}+\gamma_{t}I_{t}+\tau X_{ist}+\varepsilon_{ist}$$

The marginal impact of full-day kindergarten, *φ*, is now three separate coefficients, one for each age category. If, for example, the impact on labor force participation of full-day kindergarten is larger for women with kindergarten-age children (age 5–6) than it is for women without young children, then the estimates of *φ*_56_ will be positive.

Standard errors in a difference-in-difference regression are likely serially correlated across time, while the law change is persistent. Standard errors that do not account for this will overstate the precision so, following Bertrand, Duflo & Mullainathan (2004), we adjust by clustering the standard errors by state. We also use Census Bureau-provided individual-level probability weights when estimating the models.

## VI. First Stage

Before we discuss our main analysis, we examine the first stage of our analysis, effect of full-day kindergarten attendance on kindergarten attendance. For this analysis we use the October supplement of the CPS (1976–2017). We implement a difference-in-difference analysis, analogous to equation (1) to examine how free full-day kindergarten effect kindergarten attendance among 5- to 6-year-olds. We examine three outcomes, any kindergarten attendance, half-day attendance, and full-day kindergarten attendance. Table 3 presents the outcomes of this analysis. Coefficients should be interpreted as the average percentage point change in kindergarten attendance among 5- to 6-year-old in states with full-day kindergarten. All kindergarten attendance increases by 1.4 percentage points, a 3.2 percent increase at the baseline.^12^ Moving to compositional changes in the type of kindergarten, in states with half-day kindergarten decreases by 1.4 percentage points, an 8.2 percent change at the baseline. Full-day kindergarten attendance increases by 2.9 percentage points, an 11 percent change at the baseline. While we are not able to rule out zero in these estimates, they indicate large changes in the composition of children attending half-day and full-day kindergarten.

## VII. Results

Table 4 presents the results of our difference-in-differences model from equation (1). Difference-in-difference estimates indicate essentially no change in the labor force participation among all women. We find that women’s labor force participation decreases by 1.4 percentage points in states with full-day kindergarten but this result is not significantly different from zero.

Table 4 shows our main result, LPM estimates of Equations 2 and 3 using labor force participation as the dependent variable. In column (1) we only include an interaction of full-day kindergarten and youngest child aged 5–6 (kindergarten aged) as described in Equation 2. This allows us to compare our results using the life-cycle model described in Equation 3 to previous research that focuses only on women who are direct beneficiaries of the childcare subsidy. In column (2) we interact full-day kindergarten with age of youngest child in three categories, 0–2, 3–4, and 5–6. This specification estimates the effect of full-day kindergarten for all mothers with young children, including those with very young children who are future beneficiaries of the subsidy.

Results shown do not display the coefficients for the X vector, the main effect of year, nor the main effect of state. The coefficients on the variables in the X vector are as expected in all models.^13^ The main effect of year shows that women’s labor force participation increased between 1970–2000 then remained similar through 2015, and the main effect of state shows that Minnesota has the highest and West Virginia has the lowest rates of women’s labor force participation over the period studied.

The coefficients for the main effect of having a young child at home, θ in the equations above, in all models and for both data sets show that, in all states regardless of full-day kindergarten policies, mothers who have children ages 0–6 are less likely to be in the labor force than women without young children in the home. This effect is strongest for mothers whose youngest child is an infant, age 0–2. These women are 16.4 to 17 percentage points less likely to work than women without young children in the home in states without full day kindergarten (i.e. the coefficient on youngest child age 0–2 ranges from 0.164 to 0.17 across specifications). Mothers whose youngest child is kindergarten aged are 4.8 to 4.9 percentage points less likely to work than women without young children in the home in states without full day kindergarten.

Looking at column (1) of Table 5, the coefficient on full-day kindergarten is negative. Thus, for women without kindergarten-age children, labor force participation, on average, declined with the adoption of full-day kindergarten. This reinforces the importance of the DDD methodology. It raises the possibility that there are differential trends in women’s labor force participation in states that do and do not adopt universal full-day kindergarten. These differential trends could mask a policy effect if we did not difference out impacts by age of youngest child.

The coefficient on the interaction of full-day kindergarten and having a youngest child who is kindergarten age (ages 5–6) is positive. Estimates from Equation 1 show that mothers with kindergarten-age children responded differently to full-day kindergarten than women without kindergarten-age children, with estimated increases in labor force participation of this group by 2.9 percentage points (CPS data). In column (1) we see results that are largely consistent with previous research; when we focus on direct beneficiaries of the policy, women whose youngest child is kindergarten age, we find a small positive effect on women’s labor force participation.

The results using models with a full set of Interactions for mothers whose youngest child is anywhere from 0–6 are in column (2). The main coefficient on full-day kindergarten now references an omitted category that only includes women without young children of any age. Like the first model, the coefficient is negative, but now larger in magnitude and statistically significant.^14^ The interactions of full-day kindergarten and age of youngest child show that focusing on women whose youngest child is kindergarten age hid important detail about the impact of the policy change. Most notably, the impact of full-day kindergarten is largest for mothers whose youngest child is an infant and larger for mothers with children who are not yet kindergarten eligible than for mothers whose youngest child is kindergarten aged. This is consistent with a life-cycle theory in which women on the margin of deciding to join the labor force are more likely to be women with infants. For mothers whose youngest child is still an infant, i.e. age 0–2, the effect is 7.2 percentage points (CPS data). In column (2) we find the effect on mothers whose youngest child is kindergarten aged is 4.3 percentage points. These estimates are significantly different from each other, thus, the impact on women with infants is larger than the impact we see for mothers whose youngest child is kindergarten aged. The impact of universal free full-day kindergarten is stronger for mothers whose youngest child is an infant than it is for mothers whose youngest child is kindergarten aged.^15^

## VIII. Robustness Checks

### a. Negative Weighting

In addition to the results presented in section VII, we conducted various robustness checks.^16^ Recent advances in two-way fixed effect methods have uncovered potential issues associated with difference-in-differences methods with a staggard rollout (Goodman-Bacon 2021). The ATT, *φ*_56_, is the weighted sum of the values of labor force participation across all observations in the data set. In two-way fixed effect models, the estimator is a linear combination of treatment effects across treated units; however, when the treatment is staggered some or all the units may receive a negative weight. Under heterogeneous treatment effect, this can severely bias the estimator or even cause a reversal of sign. This paper makes use of the staggered rollout of state-mandated free full day kindergarten, meaning it’s possible that some of the treated units receive a negative weight.

**Figure 2.**
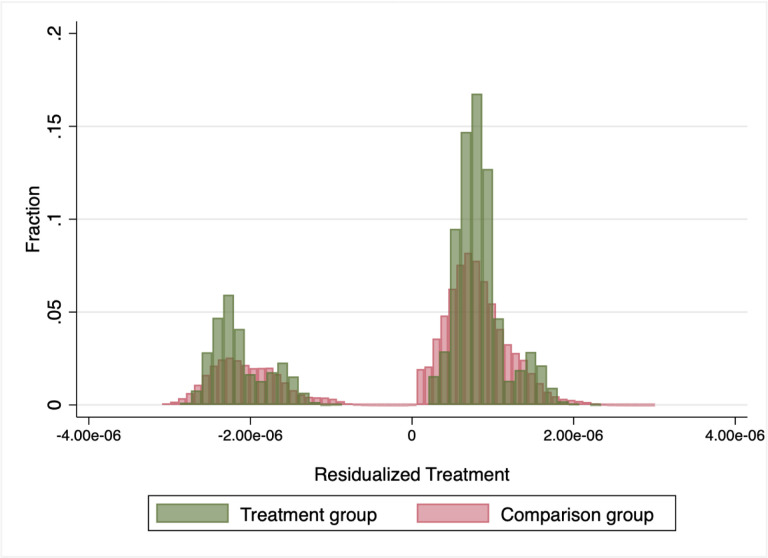
Two-Way Fixed Effects Weights, by Treatment Status Notes: Weights are calculated using the method outlined in Jakiela (2021). The treatment group is comprised of mothers with young children in state-years with universal full-day kindergarten and the comparison groups are non-mothers in those states as well as mothers with older children in state-years without universal full-day kindergarten.

To start, we examine whether any of the treated units receive a negative weight. As outlined in Jakiela (2021) the estimation weights are proportional to the residuals from a regression of the treatment on state and year-fixed effects. Figure (2) presents the histograms of the weights used to calculate the two-way fixed effects estimates on *φ*_56_. Notably, some of the treated units do, in fact, receive a negative weight. We correct for this using the estimator outlined in Borusyak, et al (2021). Our model must be adapted slightly to use the estimator outlined in Borusyak, et al (2021). Rather than estimating equation (2), we estimate equation (1) separately for each cohort. Here we compare employment outcomes of mothers with kindergarten-aged^17^ children to non-mothers and mothers whose youngest child is too old for kindergarten, before and after the introduction of universal free full-day kindergarten, in states that do and do not adopt universal full-day kindergarten. We include state, year, and age-of-youngest child fixed effect.

**Figure 3.**
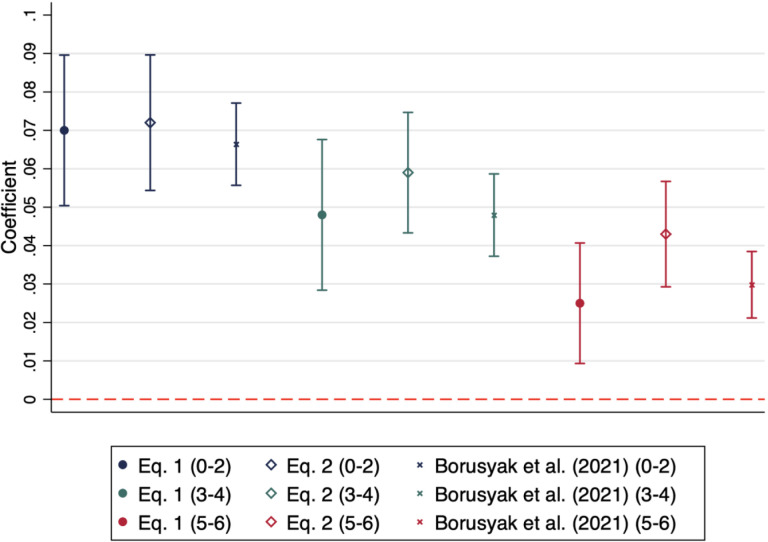
Effect of Full-Day Kindergarten on Women’s Labor Force Participation, Results by Equation Notes: This figure plots the triple-difference coefficients and confidence intervals from equation 1 (solid dots), equation 2 (hollow diamond), and using the estimator developed in Borusyak et al. (2021) (x) for each age-of-youngest-child cohort. Estimates for youngest child 0–2 are in blue. Estimates for youngest child 3–4 are in green. Estimates for youngest child 5–6 (kindergarten-aged) are in red.

Figure (3) presents the estimates from Equation 1, Equation 2, and the estimator outlined in Borusyak et al (2021) on the effect of free full-day kindergarten on maternal labor force participation by the age of her youngest child. Equation 1 and the estimator using Borusyak et al (2021) are analogous to each other, and are both presented here to ease the comparison to estimates from Equation 2. The estimates in blue display the coefficient and confidence intervals for women whose youngest child is 0–2 years. The solid blue dot graphs the coefficient obtained from estimating Equation (1) where we interact living in a state with full-day kindergarten with having the youngest child 0–2 years old. The hollow diamond graphs the coefficient from equation (2), found in Table 3. The “x” graphs the coefficient obtained using the estimator form Borusyak et al (2021). Depending on specification, estimates range from an increase in labor force participation between 6.6 and 7.2 percentage points. A test of significance reveals that there is no statistically significant difference between the coefficient obtained from Equation 1 and the coefficient obtained using the method in Borusyak et al (2021). The green estimates graph coefficients and the confidence interval for mothers whose youngest child is 3 to 4 years old. Estimates range from an increase in labor force participation between 4.8 and 5.9 percentage points. The estimates in red graph the coefficients and the confidence interval for mothers whose youngest child is 5 to 6 years old. Estimates range from an increase in labor force participation between 2.5 and 4.3 percentage points. We find no evidence that the estimates from Equation 1 are significantly different from the estimates obtained using the method in Borusyak et al (2021).

These results indicate that while some treated units receive a negative weight, it does not seriously bias our results. Under each specification, the general pattern indicates that mothers with children younger than kindergarten age see the largest gain in labor force participation relative to women with kindergarten-aged children. Women whose youngest child is 0–2 years old is the largest gain.

### b. U.S. Decennial Census Data

Next, we replicate our main analysis using the public use microdata samples (PUMS) from the U.S. decennial censuses and the American Community Survey (ACS), 1970–2015, obtained from IPUMS-USA (Ruggles et al. 2015).^18^ We elect use this data source because the CPS does not identify states individually until 1976 so the CPS data do not capture all of states adopting free full-day kindergarten. In addition to capturing the whole period of change, the samples from the decennial censuses and ACS are much larger than the CPS.

Table 6 presents these results. Column (1) presents the estimates from equation (1), column (2) presents the estimates from equation (2). Generally, the magnitude on the coefficients using the Decennial Census and ACS are larger in magnitude than those found in the CPS, but both are significant and follow a similar pattern. Comparing the estimates from equation 1, the coefficient increased from a 2.9 percentage point increase in labor force participation to a 5.4 percentage point increase. Moving to column (2) we find a similar pattern in behavior using the Decennial Census/ACS as in the CPS. Women whose youngest child is an infant are most responsive to state-mandated free full-day kindergarten, with a 9.8 percentage point increase in labor force participation.

### c. Any child kindergarten-age

Here, we estimated version equation 1 for mothers with *any* child who is kindergarten-aged (rather than just the youngest child). We find that the net impact of full-day kindergarten is near zero for mothers of any kindergarten-aged child. This is consistent with previous literature that finds an impact only when focused on the youngest child. Second, we test the robustness of equation 3 by using eldest child instead of youngest child. We find the same pattern; the net impact is still larger for mothers whose eldest child is an infant than for mothers whose eldest child is kindergarten aged. This further supports our hypothesis that mothers make key labor force decisions well before their eldest child is kindergarten aged. Important decisions are made when their eldest child is an infant, thus a life-cycle model is most appropriate. Similarly, we look at the impact for women with only one child and see the same pattern. Finally, we find the results are robust to limiting the sample to mothers (rather than all women).

### d. Heterogeneous effects

Previous research found that half-day kindergarten had a bigger impact on labor force participation of single mothers than married mothers and mothers with less than baccalaureate degree when compared to women with degrees. In this section, we estimate the effect by marital status and by highest level of education held by mothers. Table 7 reports subgroup analyses of the full model by marital status and education level using CPS data. Consistent with previous literature, we find that the positive impact of full-day kindergarten on labor force participation is larger for single women than married women and for women without a four-year college degree compared to women with a college degree. These findings are consistent with the theories posited above. Universal full-day kindergarten has the most notable impact on the labor supply decisions of women with fewer resources and this is true for women with infants and women with kindergarten aged children.

We also examine potentially heterogeneous responses to full day kindergarten over time. As shown in Table 1, the adoption of free state-wide full-day kindergarten started more than 40 years ago, and this policy continues to be adopted. It is plausible that the policy’s impact has changed over time. Women’s labor force participation as increased dramatically since the 1970’s, and 80’s when all-day kindergarten programs were first created. As women’s labor supply has increased, it’s possible the efficacy of these programs has changed. There may also be state-level differences in adoption over time. Many of the “early-adaptors” of all-day kindergarten were southern states, while more recent adaptors are from the mid-Atlantic. We estimate the full model using the CPS data for three distinct time periods within the whole period. Table 8 presents the results by the three time periods (1976–1989, 1990–1999, 2000–2015). In each time period, we find significant impacts of free full-day kindergarten for women with children aged six and under, with the largest impacts for women with infants and toddlers. The effect of the policies is larger in magnitude and significantly different between 1976–1999 than following 2000.

### e. Event-Study

For robustness, we also performed a pseudo-event-study analysis accounting for the number of years prior to the adoption of universal full-day kindergarten and the number of years following adoption using the cross-sectional CPS data. Like the results from the DDD models, we find consistent and statistically significant effects following adoption on maternal labor force participation, and the effects continue for years past adoption. We omitted event-year zero, when full-day kindergarten legislation was passed. We estimate changes in average labor force participation for mothers whose youngest child is 0–2 (3–4, or 5–6) in that event year, relative to the adoption of full-day kindergarten.

**Figure 4.**
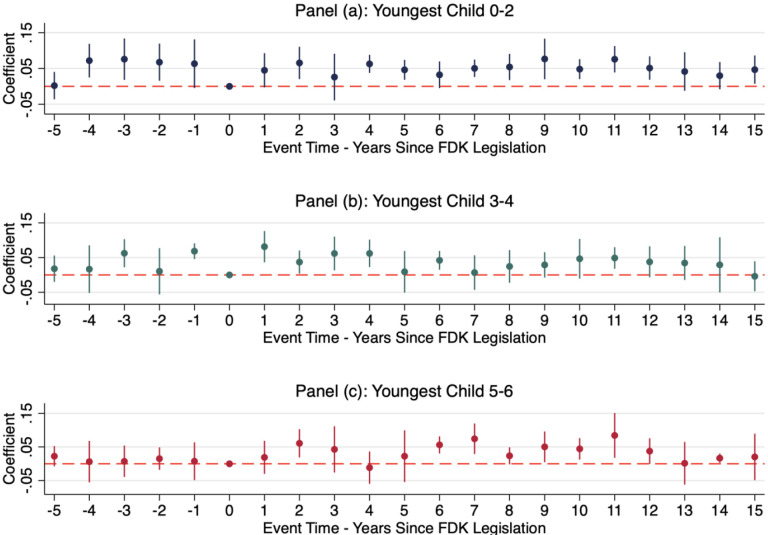
Event Study Estimates of the effect of full-day kindergarten on women’s labor force participation Notes: Results are from CPS data, and the sample includes all women ages 18–55. The models also include the following variables: age, age-squared, college-age indicator, married indicator, race, education, number of children in household, number of adults in the household, non-labor income, non-labor income squared, urban residence, state fixed effects, and year fixed effects. Robust standard errors clustered at the state level. This figure plots the triple-difference coefficients and confidence intervals of event study estimates of the effect of full-day kindergarten on

Figure 4 graphs event-study coefficients. Panel (a) depicts the change in labor force participation for mothers of 0–2-year-olds in each year, relative to the year full-day kindergarten was passed. There is some evidence that women with children 0–2 were increasing their labor force participation prior to the introduction of full-day kindergarten. We therefore cannot fully rule out that women with 0–2-year-olds were differentially changing their labor supply prior to the adoption of full-day kindergarten using this design. In the year following full-day kindergarten passage, mothers of 0–2-year-olds were 4.4 percentage points more likely to be in the labor force, this effect increases in the following year to 6.5 percentage points. This effect stays positive and significant in most years after the state-level adoption of full-day kindergarten.

Panel (b) depicts the change in labor force participation for mothers of 3–4-year-olds. We find little evidence that mothers of 3–4-year-olds were changing their labor supply prior to the adoption of full-day kindergarten. Pseudo-event-study results indicate that mothers of 3–4-year-olds increase their labor force participation. In the year following the implementation of full-day kindergarten mothers of 3–4-year-olds are 8.1 percentage points more likely to be in the labor force. This effect decreases in size over time but stays positive and significant in most years.

Panel (c) depicts the change in labor force participation for mothers of 5–6-year old’s labor force participation. We find little evidence that mothers of 5–6-year-olds were changing their labor supply prior to the state-level adoption of full-day kindergarten. In the year following the passage of full-day kindergarten, mothers of kindergarten-aged children were 1.9 percentage points more likely to be in the labor force. This effect stays positive and significant in most years following the adoption of full-day kindergarten.

### f. Annual Hours Worked

Finally, we examine the effect of full day kindergarten on mothers’ annual hours worked. With the strong effects of universal free full-day kindergarten on the labor force participation of mothers with young children, it is likely that the intensive margin of labor supply will also be affected by this policy. Table 9 presents the results for yearly hours worked for employed women. Column (1) presents the results from the CPS data, which is preferred for measurement consistency, and the results in column (2) are from the decennial census/ACS data. The results from both data sets are consistent with the findings for the extensive margin; universal free full-day kindergarten increases the hours employed mothers work, and the effect is the largest for women with young children. The results in column (1) show that the introduction of full-day kindergarten increases the hours women with a youngest child age 0–2 or 3–4 by approximately 90 hours per year. Women with children kindergarten-age also experience an increase in hours work per year (73 hours per year). These results from the CPS are slightly larger than what we find using the decennial censuses/ACS, which show about a 60 hour increase in work hours for mothers of children under age six.

## IX. Discussion

Our results provide new insight into the effect of schooling on women’s labor supply. Our results mirror previous research on the impact of kindergarten on mothers whose youngest child is kindergarten aged. Importantly, however, we also show that kindergarten, or more specifically a state mandate for free universal full-day kindergarten, has a larger impact on mothers whose youngest child is an infant or pre-kindergarten age than it does on mothers whose youngest child is kindergarten aged. While these mothers are not the direct beneficiaries of the expanded childcare, this result is consistent with a life-cycle model of women’s labor supply where women at the margin of labor force attachment are mothers with very young children. Mothers whose youngest child is kindergarten aged have already made key decisions about labor force attachment and thus are less likely to be impacted by policy changes.

We implement a triple difference estimator, which helps to mitigate endogeneity between women’s labor supply decisions and the presence of children in their household. It cannot, however, address issues that arise if women make changes to their fertility decisions *because* they have access to full day kindergarten. If women do change their lifetime fertility, it may ambiguously affect out results, depending on how it interacts with mothers’ fertility choices. If the presence of full day kindergarten increases the number of children a woman has, it may decrease her labor supply, at least in the short run (Angrist & Evans, 1996; Cools et al., 2017). Conversely, if women reduce their lifetime fertility due to the change, it may increase labor supply.

Policymakers will find these results relevant for decisions about full-day kindergarten as well as other childcare-related policies such as pre-kindergarten and Head Start expansions, childcare subsidies/tax credits, and parental leave. To estimate a full accounting of the impact the policy has, it is important to look not only at the direct beneficiaries but also the indirect or future beneficiaries who are making crucial decisions about when to return to work after the birth of a child. Omitting the benefit for mothers of younger children will undercount the impact of full-day kindergarten legislation on women’s labor force participation and lead to suboptimal policy choices. Our findings also point to the importance of stable and predictable childcare for parents. The expectation of future full-day kindergarten enables mothers to return or remain attached to the labor market without interruption. Just as families make important decisions about where to buy a house so that they can take advantage of future schooling options (e.g. Black 1999), mothers make important decisions about work based on future availability of full-day kindergarten.

## Figures and Tables

**Table 1.** States adopting free full-day kindergarten by year

State	Year
Alabama	1973
Washington DC	1978
North Carolina	1978
Mississippi	1986
Louisiana	1990
South Carolina	1996
West Virginia	1996
Arkansas	2002
Maryland	2002
Delaware	2005
Oklahoma	2013

Source: Education Commission of the States (https://www.ecs.org), Children’s Defense Fund (http://www.childrensdefense.org/), and review of news and legal reports.

**Table 2.** Summary characteristics for women ages 18–55

	All	Full Day	Not Full Day
Age	35.83	35.82	35.84
Married	0.583	0.582	0.583
White, non-Hispanic	0.702	0.703	0.702
Black, non-Hispanic	0.128	0.238	0.110
Other	0.170	0.059	0.188
Less than high school	0.132	0.141	0.131
High school degree or GED	0.343	0.357	0.341
Some college	0.283	0.275	0.284
College or advanced degree	0.241	0.227	0.244
Number of adults in household	2.080	2.064	2.083
Number of children in household	1.128	1.115	1.130
Children age 0–18	0.509	0.517	0.508
Youngest child age 0–2	0.132	0.130	0.132
Youngest child age 3–4	0.066	0.066	0.066
Youngest child age 5–6	0.055	0.057	0.055
Youngest child age 7–8	0.050	0.051	0.050
Youngest child age 9–18	0.207	0.213	0.206
Non-labor income	36,164	33,081	36,676
Urban residence	0.813	0.689	0.833
Number of observations	1,743,739	266,251	1,477,488

Source: Authors’ calculations from Current Population Survey Annual Social and Economic Supplement yearly data 1978–2015 (Flood et al 2015). Sample is restricted to women ages 18–55. ‘All Day K’ indicates states that ever adopted free all day kindergarten, ‘Not All Day K’ indicates states that never adopted all day kindergarten.

**Table 3.** The effect of full-day kindergarten on children’s kindergarten attendance

	Any Kindergarten	Half-Day	Full-Day
Full-Day Kindergarten	0.014 (0.022)	−0.014 (0.032)	0.029 (0.052)
Observations	112,543	112,543	112,543
Pseudo R-squared	0.029	0.077	0.058

Notes; The samples includes all children ages 5–6 from the October CPS Sample. The models also include the following variables: Race, state fixed effects, and year fixed effects. The reported coefficients are the estimated change in probability of kindergarten participation associated with a discrete change in the independent variable, calculated at the mean of the sample. Robust standard errors clustered at the state and year level are reported in the parenthesis.

* p<.05;

** p<.01;

*** p<.001

**Table 4.** The effect of full-day kindergarten on women’s labor force participation (DiD)

	(1)
Full-Day Kindergarten	−0.014 (0.009)
Observations	1,743,739
Pseudo R-squared	0.084

Notes: The samples includes all women ages 18–55. The models also include the following variables: age, age-squared, college age indicator, married indicator, race, education, number of children in household, number of adults in household, non-labor income, non-labor income squared, urban residence, state fixed effects and year fixed effects. The reported coefficients are the estimated change in probability of labor force participation associated with a discrete change in the independent variable, calculated at the mean of the sample. Robust standard errors clustered at the state and year level are reported in the parenthesis.

* p<.05;

** p<.01;

*** p<.001

**Table 5.** The effect of full-day kindergarten on women’s labor force participation

	(1)	(2)
Full-Day Kindergarten	−0.015 (0.009)	−0.028 ** (0.009)
Full-Day Kindergarten*Youngest Child Age 0–2		0.072 *** (0.009)
Full-Day Kindergarten*Youngest Child Age 3–4		0.059 *** (0.008)
Full-Day Kindergarten*Youngest Child Age 5–6	0.029 *** (0.006)	0.043 *** (0.007)
Youngest Child Age 0–2	−0.164 *** (0.005)	−0.170 *** (0.005)
Youngest Child Age 3–4	−0.088 *** (0.006)	−0.094 *** (0.006)
Youngest Child Age 5–6	−0.048 *** (0.005)	−0.049 *** (0.005)
Observations	1,743,739	1,743,739
R-squared	0.098	0.099

Notes: The samples includes all women ages 18–55. The models also include the following variables: age, age-squared, college age indicator, married indicator, race, education, number of children in household, number of adults in household, non-labor income, non-labor income squared, urban residence, state fixed effects and year fixed effects. Robust standard errors clustered *p<.05; **p<.01; ***p<.001

**Table 6.** The effect of full-day kindergarten on women’s labor force participation (Decennial Census/ACS)

	(1)	(2)
Full-Day Kindergarten	−0.027 (0.014)	−0.044 ** (0.014)
Full-Day Kindergarten*Youngest Child Age 0–2		0.098 *** (0.013)
Full-Day Kindergarten*Youngest Child Age 3–4		0.079 *** (0.010)
Full-Day Kindergarten*Youngest Child Age 5–6	0.054 *** (0.006)	0.071 *** (0.008)
Youngest Child Age 0–2	−0.170 *** (0.006)	−0.178 *** (0.007)
Youngest Child Age 3–4	−0.096 *** (0.007)	−0.103 *** (0.007)
Youngest Child Age 5–6	−0.056 *** (0.006)	−0.058 *** (0.006)
Observations	12,443,157	12,443,157
R-squared	0.124	0.125

Notes: The samples includes all women ages 18–55. The models also include the following variables: age, age-squared, college age indicator, married indicator, race, education, number of children in household, number of adults in household, non-labor income, non-labor income squared, urban residence, state fixed effects and year fixed effects. Robust standard errors clustered *p<.05; **p<.01; ***p<.001

**Table 7.** The effect of full-day kindergarten on women’s labor force participation by marital status and educational attainment

	Marital Status		Educational Attainment	
	Married	Single	No College Degree	College Degree
	(1)	(2)	(5)	(6)
All Day Kindergarten	−0.030 * (0.012)	−0.017 * (0.008)	−0.035 ** (0.010)	−0.016 * (0.006)
All Day Kindergarten*Child Age 0–2	0.050 *** (0.009)	0.084 *** (0.015)	0.078 *** (0.010)	0.054 ** (0.019)
All Day Kindergarten*Child Age 3–4	0.045 *** (0.010)	0.061 *** (0.011)	0.066 *** (0.009)	0.041 ** (0.013)
All Day Kindergarten*Child Age 5–6	0.028 *** (0.006)	0.049 *** (0.012)	0.046 *** (0.008)	0.034 ** (0.010)
Child Age 0–2	−0.204 *** (0.006)	−0.110 *** (0.009)	−0.182 *** (0.007)	−0.139 *** (0.005)
Child Age 3–4	−0.129 *** (0.007)	−0.038 *** (0.007)	−0.100 *** (0.007)	−0.085 *** (0.007)
Child Age 5–6	−0.081 *** (0.006)	−0.006 (0.006)	−0.056 *** (0.006)	−0.039 *** (0.005)
Observations	1,057,379	686,360	1,334,358	409,381
R-squared	0.102	0.105	0.070	0.082

Notes: The results are from CPS data, and the sample includes all women ages 18–55. The models also include the following variables: age, age-squared, college age indicator, married indicator, race, education, number o children in household, number of adults in household, non-labor income, non-labor income squared, urban residence, state fixed effects and year fixed effects. Robust standard errors clustered at the *p<.05; **p<.01; ***p<.001

**Table 8.** The effect of full-day kindergarten on women’s labor force participation by time

	1976–1989	1990–1999	2000–2015
	(1)	(2)	(3)
All Day Kindergarten	−0.044 *** (0.008)	−0.004 (0.019)	−0.027 *** (0.005)
All Day Kindergarten*Child Age 0–2	0.075 *** (0.013)	0.074 *** (0.010)	0.043 *** (0.010)
All Day Kindergarten*Child Age 3–4	0.067 *** (0.014)	0.080 *** (0.009)	0.029 * (0.011)
All Day Kindergarten*Child Age 5–6	0.045 ** (0.013)	0.043 ** (0.015)	0.028 *** (0.007)
Child Age 0–2	−0.204 *** (0.011)	−0.170 *** (0.008)	−0.121 *** (0.005)
Child Age 3–4	−0.175 *** (0.013)	−0.105 *** (0.008)	−0.053 *** (0.004)
Child Age 5–6	−0.081 *** (0.011)	−0.055 *** (0.008)	−0.021 *** (0.004)
Observations	531,128	796,579	831,593
R-squared	0.130	0.116	0.081

Notes: The results are from CPS data, and the sample includes all women ages 18–55. The models also include the following variables: age, age-squared, college age indicator, married indicator, race, education, number o children in household, number of adults in household, non-labor income, non-labor income squared, urban residence, state fixed effects and year fixed effects. Robust standard errors clustered at the state level are *p<.05; **p<.01; ***p<.001

**Table 9.** The effect of full-day kindergarten on annual weeks worked Multiplied by usual weekly hours, regression results

	(1)
Full-Day Kindergarten	−31.99 ** (10.21)
Full-Day Kindergarten*Youngest Child Age 0–2	91.91 *** (10.67)
Full-Day Kindergarten*Youngest Child Age 3–4	91.60 *** (13.33)
Full-Day Kindergarten*Youngest Child Age 5–6	73.65 *** (14.14)
Youngest Child Age 0–2	−239.42 *** (8.86)
Youngest Child Age 3–4	−161.19 *** (9.85)
Youngest Child Age 5–6	−152.48 *** (9.81)
Observations	1,319,039
R-squared	0.134

include the following variables: age, age-squared, college age indicator, married indicator, race, education, number of children in household, number of adults in household, non-labor income, non-labor income squared, urban residence, state fixed effects and year fixed effects. Robust standard errors clustered at the state level are reported in the parenthesis.

* p<.05;

** p<.01;

*** p<.001

## Footnotes

1 It is possible that a mother with kindergarten aged children would experience an income effect, thus causing her to reduce weekly hours worked to engage in home production. In this paper we assume that substitution effect is stronger than the income effect and thus mothers will work more hours. Our findings are consistent with this assumption.

2 There is a substantial literature that documents a “time-out” penalty for mothers (Lundberg & Rose, 2000; Aisenbrey, Evertsson, & Grunow, 2009; Staff & Mortimer, 2012; Hotchkiss et al. 2017). A forthcoming paper by Katie Genadek, Philip Pendergast, and Kristine West (presented at the Western Economic Conference) finds evidence of a non-linear relationship between years out of the labor market and wage penalty.

3 In a supplementary analysis, we examine the effect of having *any* kindergarten-aged child in the home. We find that the net impact of full-day kindergarten is near zero. This result is consistent with the main results in this paper, as well as with previous literature that finds little or no impact when focusing on women with kindergarten aged children.

4 The typical half-day program is 3 hours. Source: https://naspcenter.org/assessment/kindergarten-full-versus-half-day.

5 Tennessee adopted full-day kindergarten legislation in 2010 but is excluded from our analysis. In Tennessee, the definition of a “full day” is four hours (Education Commission of the States 2016). All other full-day kindergarten states define a full day as at least five hours. Additionally, there are four states (Alaska, Florida, Georgia and Hawaii) without all-day kindergarten legislation where minimum hours for kindergarten that meet or exceed Tennessee’s four hour requirement. Robustness checks that include Tennessee are available upon request.

6 Other states with high full-day kindergarten take-up (in the absence of a state mandate) include Hawaii, Kansas, Kentucky, Iowa, Maine, Michigan, Minnesota, Missouri, Montana, Nebraska, South Dakota and Vermont.

7 We argue using state-level has advantages over county level variation. Using county-level variation can be problematic as parents can enroll their children across county-lines in some states. Conversely, it’s much more arduous to cross state-lines for school enrollment.

8 The ACS replaced the long-form survey used from the U.S. decennial census following the 2000 census.

9 For the analysis we use the following PUMS from the decennial census long forms: 1970 1% state samples, 1980 5% sample, 1990 5% sample, and 2000 5% sample. We also use the ACS 1% PUMS from 2005, 2010, and 2015.

10 Many studies use ages 25–55 as the prime working age, but we use a wider age range for our analysis because we are particularly interested in labor supply for women with young children.

11 We also estimated the effect of universal full-day kindergarten on fathers’ labor supply. The analyses suggest that the policy did not impact father’s labor force participation or hours worked per year. Results available from the authors.

12 Forty-three percent of children in states without state-mandated full-day kindergarten attend kindergarten. Among children who do attend kindergarten, seventeen percent attend half-day kindergarten and twenty-six percent attend full-day kindergarten.

13 Full regression output is available upon request. The coefficients on the quadratic in age are as expected, in both samples, women are more likely to work as they get older but this increase diminishes (i.e. the linear term is positive but the squared term is negative). Additionally, even after controlling for the continuous variable that measures age, women who are aged 18–22 are less likely to work, presumably because they are more likely to be in school full time. Non-labor income, which includes spousal income, decreases the probability that a woman will be in the labor force, although the coefficient on non-labor income squared is positive suggesting that at high incomes, this trend reverses. This is consistent with women at the lower end of the household income distribution facing negative net earnings after paying for childcare and thus deciding to opt out of the labor force while higher earning households are more likely to return to work after the birth of a child.

14 We consider two hypotheses for why mothers with older children and non-mothers may experience declines in labor force participation after the introduction of full-day kindergarten. First, universal free full-day kindergarten may displace childcare workers. Full-day kindergarten will increase the demand for licensed kindergarten teachers and decrease the demand for in-home and center-based childcare. This is an industry that is almost entirely female and may employ a number of women who are otherwise on the margin of labor force participation. Secondly, there may be other general equilibrium effects. If full-day kindergarten increases labor force participation for mothers of young children, these workers may displace other workers, and if the displacement happens in female-dominated industries, the displacement may disproportionately impact women.

15 We examine the effect of full-day kindergarten for women with any kindergarten aged child. Our results under this specification are consistent with findings in the previous literature. The net effect of women with kindergarten aged child is near zero.

16 All robustness results are available upon request from authors.

17 We also estimate models for a youngest child 0–2 and 3–4.

18 The ACS replaced the long form survey used from the U.S. decennial census following the 2000 census.
